# Ultrasonography diagnosis of dumbbell−shaped C5 cervical schwannoma: a case report and literature review

**DOI:** 10.3389/fonc.2025.1490713

**Published:** 2025-06-05

**Authors:** Kailin Yu, Jingsen Chen, Minqiang Pan, Kanlun Xu, Pintong Huang, Yucong Peng

**Affiliations:** ^1^ Department of Ultrasound in Medicine, The Second Affiliated Hospital, Zhejiang University School of Medicine, Hangzhou, Zhejiang, China; ^2^ Department of Neurosurgery, The Second Affiliated Hospital, Zhejiang University School of Medicine, Hangzhou, Zhejiang, China; ^3^ Department of Pathology, The Second Affiliated Hospital, Zhejiang University School of Medicine, Hangzhou, Zhejiang, China

**Keywords:** ultrasonography, dumbbell-shaped tumor, cervical schwannoma, spinal tumor, schwannoma, imaging modalities

## Abstract

**Introduction and importance:**

Dumbbell-shaped C5 schwannomas are rare lesions that involve both intraspinal and extra-spinal communicating compartments. Early diagnosis and complete resection are of great significance in the treatment of dumbbell-shaped cervical schwannomas.

**Case presentation:**

The authors present a case of a 69-year-old female patient who has detected a palpable cervical mass for the preceding year. The patient had not sought medical consultation until pain and numbness in her right upper limb occurred over the span of two weeks. The initial ultrasound examination revealed a C5 dumbbell-shaped schwannoma with size of 6.07 cm * 2.54 cm, which expediting a timely magnetic resonance imaging (MRI) examination and subsequent surgical intervention for the patient.

**Clinical discussion:**

Despite the majority being benign, spinal schwannomas are characterized by an insidious onset, with patients typically presenting for medical attention at a stage when they exhibit severe symptoms, including but not limited to cervical and shoulder pain, paresthesia of the limbs, and motor weakness. A definitive diagnosis is often confirmed through computed tomography (CT) or MRI. Regrettably, an extended disease duration can occasionally result in a degree of neurological dysfunction that is refractory to complete recovery. Notably, ultrasonography, as an accessible imaging modality, is equally capable of visualizing critical structures within and surrounding the spinal canal, facilitating the early detection and diagnosis of occult spinal schwannomas, particularly for dumbbell-shaped schwannomas involved intraspinal and extra-spinal compartments.

**Conclusion:**

This study highlights the relevance of ultrasonography for the initial evaluation, interdisciplinary and coordinated work in the management of spinal tumor.

## Introduction

Schwannomas, which originate from the peripheral nervous system, are benign neoplasms (1). However, the occurrence of large, dumbbell-shaped schwannomas involving both the intradural and extradural spaces is exceedingly rare in clinical practice and is typically diagnosed using magnetic resonance imaging (MRI) or computed tomography (CT). With the accumulation of experience and advancements in technology, the utility of ultrasonography in diagnosing schwannomas involving both the intraspinal and extra-spinal compartments has been increasingly recognized. Hereby, we present a case of a cervical dumbbell-shaped schwannoma initially diagnosed via ultrasonography and reviews the relevant literature regarding its diagnosis and management process.

## Case presentation

A 69-year-old female of Han Chinese ethnicity, with no history of cervical trauma or prior spinal surgery, presented with a history of a self-palpated, soft, egg-sized mass in the right cervical region one year prior, which was initially disregarded. It was not until the recent two weeks, when she noticed an increase in the size of the mass accompanied by pain in the right upper limb, that she presented to medical help. Physical examination revealed a 2 cm diameter, soft mass in the right supraclavicular region, with concomitant hypoesthesia of the right upper limb.

**Figure 1:**
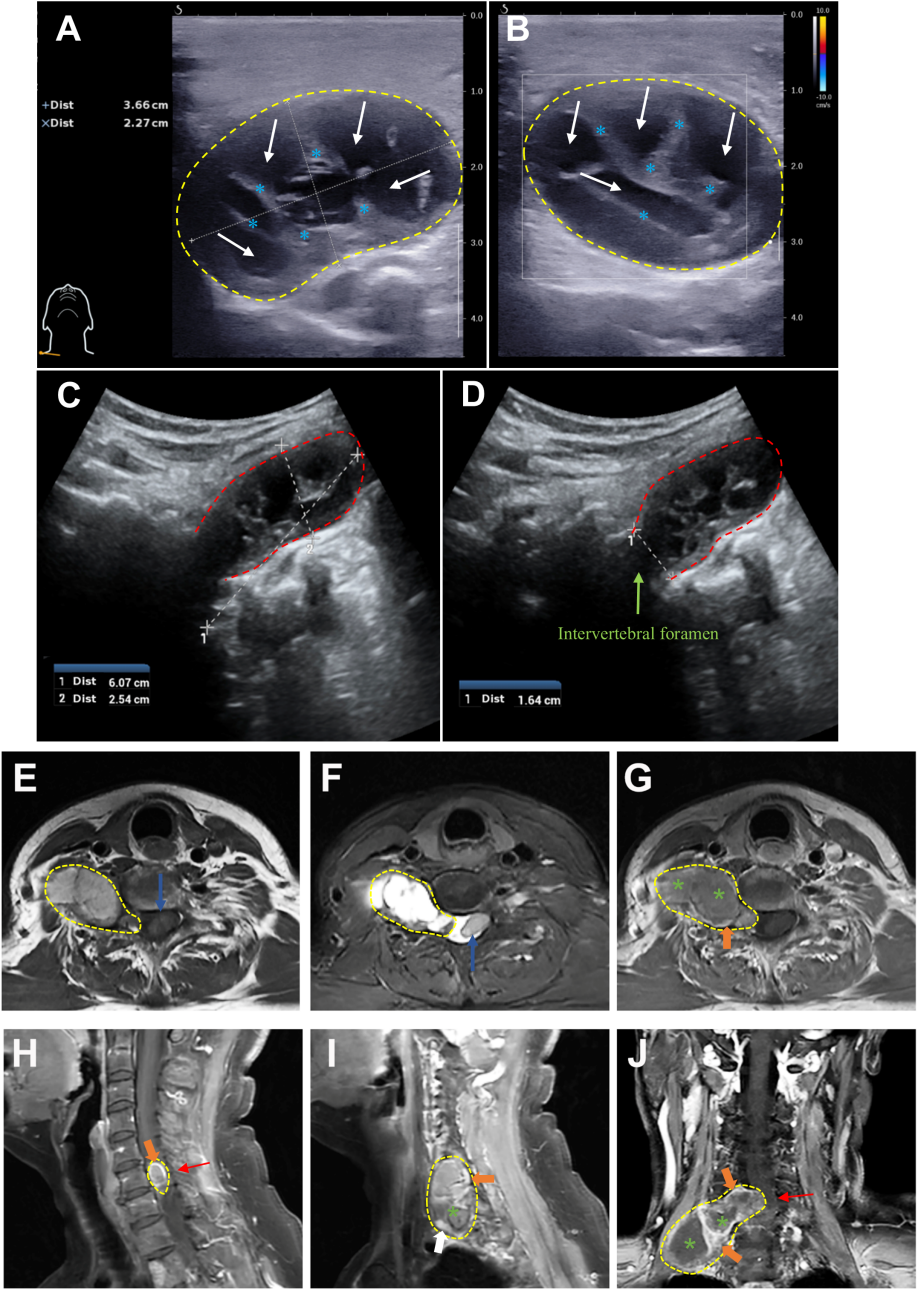
The preoperative ultrasonographic and MRI images of a giant dumbbell-shaped C5 cervical schwannoma in a 69-year-old female. **(A, B)** Superficial probe imaging (12 MHz): A mixed cystic-solid mass (3.66 cm × 2.27 cm, yellow dashed outline) is visualized in the right supraclavicular region. The solid component exhibits heterogeneous echogenicity with linear hyperechoic structures (blue asterisk), while the cystic area displays anechoic fluid content (white arrow). Color Doppler flow imaging (CDFI) revealed no significant vascularity within the solid component. **(C, D)** Transabdominal probe imaging (5 MHz): A cystic-solid mass (6.07 cm × 2.54 cm, red dashed outline) extends from the C5 intervertebral foramen (green arrowhead) into the paravertebral space. **(E)** Axial T1-weighted imaging (T1WI) and **(F)** T2-weighted imaging (T2WI) reveal a heterogeneous dumbbell-shaped mass (predominantly isointense on T1WI and hyperintense on T2WI) extending from the C5 vertebral level into the paravertebral space. The intraspinal component causes spinal canal stenosis and displacement of the spinal cord (blue arrow). **(G)** Axial, **(H, I)** sagittal, and **(J)** coronal contrast-enhanced T1WI sequences demonstrate peripheral and septal enhancement (orange arrowheads) with non-enhancing cystic regions (green asterisk). Significant spinal cord compression (red arrow) and transforaminal extension of the tumor through the right C5 intervertebral foramen into the thoracic inlet are observed. The imaging characteristics are consistent with a cystic schwannoma involving both intraspinal and extra-spinal compartments (yellow dashed line).

The patient initially underwent ultrasonography, which, using a superficial probe, identified a mixed cystic and solid mass measuring approximately 3.66 cm * 2.27 cm in the right supraclavicular area, with clear boundaries and no significant vascular flow within the solid component observed on Color Doppler flow imaging (CDFI) (**Figures 1A, B**). Furthermore, a “deeply hidden” mass was delineated by utilizing a transabdominal probe. Sonographic findings included a predominantly cystic mass, 6.07 cm *2.54 cm in dimensions, with its orifice at the C5 vertebral foramen, measuring approximately 1.64 cm in diameter. The mass exhibited clear demarcation and internal fibrous echoes, and lacked significant vascular flow on CDFI (**Figures 1C, D**), indicating the diagnosis of a giant dumbbell−shaped C5 cervical schwannoma with cystic degeneration.

Subsequent cervical magnetic resonance imaging (MRI) demonstrated a lesion extending from the level of the fifth cervical vertebra within the spinal canal, causing spinal stenosis and displacement of the spinal cord, with the main body of the lesion extending laterally and inferiorly through the intervertebral foramina and intervertebral spaces, reaching down to the level of the thoracic inlet, exhibiting layered signal intensity in its lower part, with no enhancement post-contrast, and measuring approximately 6.0cm * 2.5cm in its largest coronal dimension (**Figures 1E–J**). The MRI diagnosis indicated a schwannoma with cystic degeneration, which is consistent with the findings from ultrasonography.

**Figure 2:**
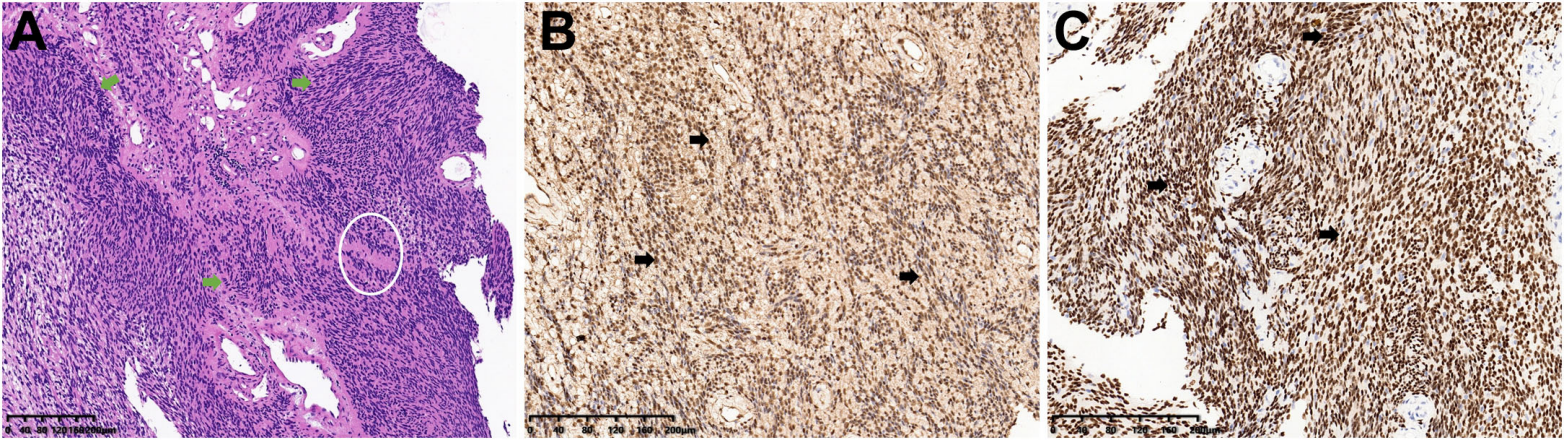
Histopathological confirmation of schwannoma. **(A)** Hematoxylin and eosin (H&E) staining demonstrated a typical schwannoma Antoni A area with palisading spindle cells (green arrow) and Verocay body (white circle). **(B, C)** Immunohistochemistry demonstrated strong S-100 **(B)** and SOX-10 **(C)** protein positivity (brown staining, black arrow), confirming schwann cell origin.

**Figure 3:**
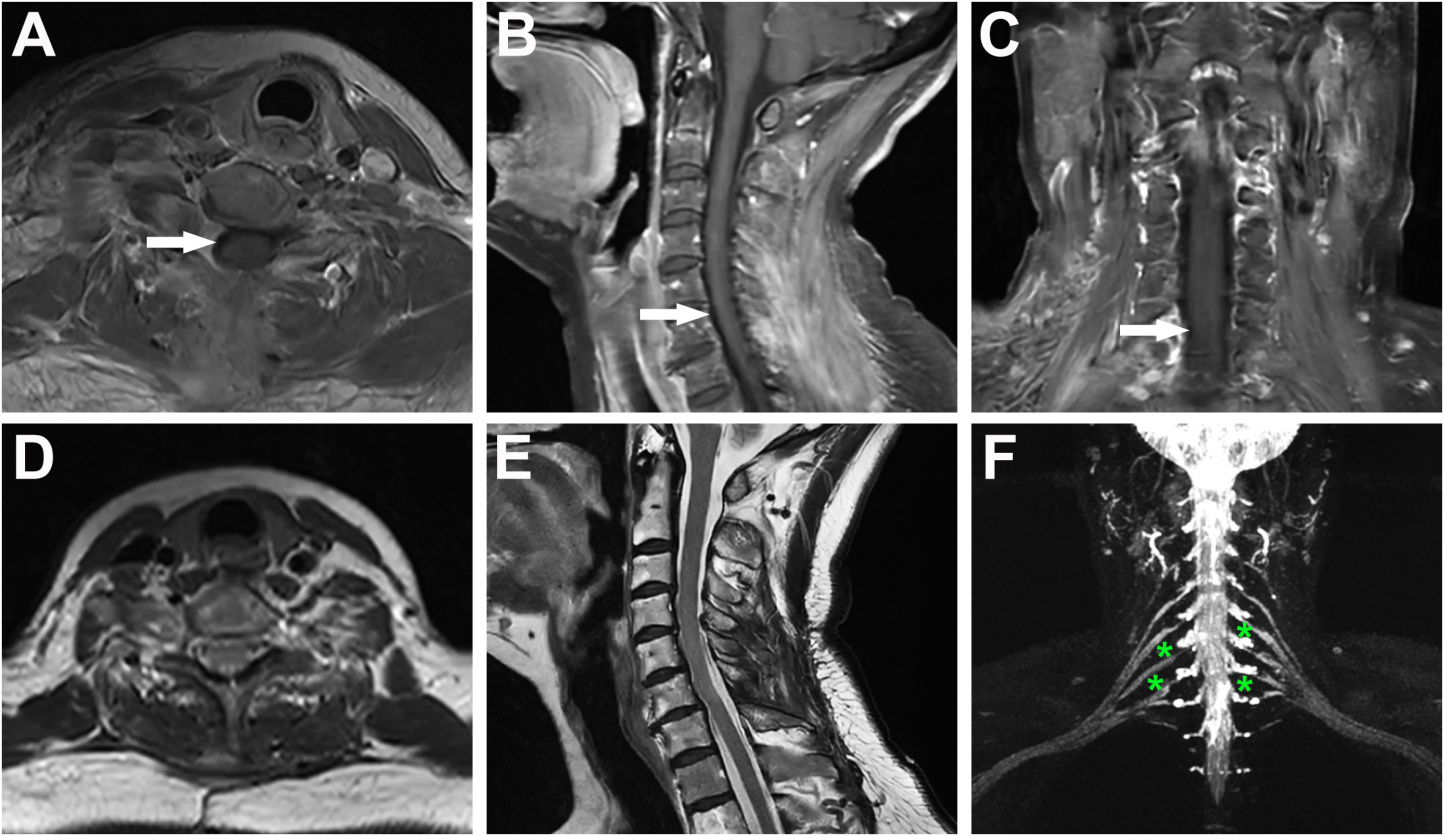
Postoperative MRI evaluation at 6-month and 1-year follow-up confirmed complete tumor resection without residual or recurrent lesions. **(A–C)** Contrast-enhanced cervical MRI at 6 months post-surgery (axial, sagittal, and coronal views) demonstrates relief of spinal cord compression (white arrows). The absence of tumor enhancement confirms complete resection without residual or recurrent lesions. **(D–F)** Contrast-enhanced brachial plexus MRI at 1-year follow-up (axial, sagittal, and nerve reconstruction views) reveal no tumor recurrence. The bilateral brachial plexuses are clearly delineated (green asterisks), indicating preserved neural integrity and absence of iatrogenic injury.

The patient underwent surgical resection of the intradural and extradural components of the tumor via a posterior approach on November 30, 2022 and lateral approach on March 8, 2023 with general anesthesia and intraoperative neuromonitoring, respectively. No significant cerebrospinal fluid leakage or post-operation infection occurred. And histopathological examination, with typical Antoni A areas and strong S-100 and SOX-10 expression, confirmed a diagnosis of schwannoma (**Figure 2**). Contrast-enhanced MRI of the neck and brachial plexus was performed at 6-month and 1-year follow-up. The imaging demonstrated complete resolution of spinal cord compression, absence of residual or recurrent tumor signals, and clear delineation of bilateral brachial plexuses, suggesting a successful tumor resection without residual/recurrence or iatrogenic brachial plexus injury (**Figure 3**). Notably, the patient rated her preoperative right upper limb pain as 7/10 on the VAS, with numbness severely affecting her ability to hold objects (NDI score: 38/50). During the postoperative interviews at the 6-month and 1-year follow-up, the patient reported sustained resolution of right upper limb pain (with pain score decreasing from 7/10 to 2/10 on the VAS) and restored grip strength (with NDI score decreasing from 38/50 to 10/50). She further emphasized a significant improvement in quality of life, with complete resolution of symptoms and no residual functional limitations, describing “a *profound sense of relief, as though a persistent burden had been removed*” (with PGIC: ‘very much improved’, **Table 1**). These subjective outcomes align with objective imaging findings, underscoring the efficacy of the intervention.

**Table 1:** Timeline of patient care and interventions.

Time Point	Clinical Event/Intervention	Key Findings/Outcomes
1-0ct-21	Patient self-palpates a soft, egg-sized mass in the right cervical region.	No medical consultation sought initially.
9-0ct-22	Development of progressive right upper limb pain and numbness.	Prompted medical evaluation. Pain as 7/10 on the VAS, affected grip strength with NDI score 38/50.
Initial presentation 10-0ct-22	Superficial ultrasound (12 MHz probe).	Detected a 3.66 cm × 2.27 cm mixed cystic-solid mass in the right supraclavicular area.
Follow-up imaging 10-0ct-22	Deep ultrasonography (5 MHz probe) and cervical MRI.	Confirmed a 6.07 cm × 2.54 cm dumbbell-shaped schwannoma extending from C5 foramen.
30-Nov-22	First-stage surgery: Posterior approach for intradural tumor resection.	Removed spinal canal component; no CSF leakage or infection.
8-Mar-23	Second-stage surgery: Lateral approach for extradural tumor resection.	Complete removal of paravertebral component without complications
6-month follow-up	Postoperative MRI and clinical assessment.	No residual tumor; pain score reduced to 2/10 on VAS; restored grip strength with 10/50 on NDI score, PGIC is very much improved
1-year follow-up	Final MRI and functional evaluation.	Sustained tumor-free status; full restoration of daily activities (NDI 10/50), PGIC: “very much improved”

VAS, visual analog scale; NDI, neck disability index; CSF, cerebrospinal fluid; PGIC, patient global impression of change.

## Discussion

The current case underscores three pivotal clinical insights. Firstly, ultrasonography served as an important modality for detecting a large spinal schwannoma, revealing well-defined margins, cystic degeneration, and minimal vascularity despite the absence of the classic “tail sign,” thereby highlighting its utility in early diagnosis of paraspinal masses. Subsequently, MRI played an indispensable role in delineating the tumor’s spatial relationship with neurovascular structures and confirming complete resection, demonstrating the complementary synergy between imaging modalities. Furthermore, the tumor’s extensive extra-spinal extension (>4 cm) could choose a two-stage posterior and lateral surgical approach, emphasizing the importance of tailored strategies based on tumor characteristics and anatomical complexity, as guided by the Azsazuma classification. These findings collectively reinforce ultrasonography’s diagnostic value, the necessity of multimodal imaging integration, interdisciplinary work and the need for individualized surgical planning in managing complex spinal schwannomas.

Schwannomas are benign peripheral nerve sheath tumors that arise from the Schwann cells enveloping myelinated nerve fibers. They are typically sporadic and exhibit a minimal malignant potential (1, 2). These tumors can occur anywhere along the neuroaxis but show a predilection for the head, neck, and spinal canal. Intraspinal schwannomas are the most common extramedullary, intradural neoplasms, comprising 25-30% of spinal canal tumors, with a heightened frequency in the cervical and thoracic spine (3, 4). Microscopically, schwannomas demonstrate a biphasic pattern, consisting of the densely packed Antoni A areas characterized by spindle-shaped cells in a palisaded arrangement, and the less cellular Antoni B areas with a myxoid matrix and verocay bodies (5). These neoplasms are encapsulated and may present as ovoid or lobulated masses. Larger tumors can exhibit calcification, cystic degeneration, or hemorrhage. The majority of spinal schwannomas are situated intradurally, with a subset extending extradurally or even protruding through the intervertebral foramina, resulting in the classic dumbbell-shaped appearance and potential enlargement of the neural foramina (6). Originating predominantly from the dorsal nerve roots, schwannomas can lead to chronic spinal cord compression, marked by discernible indentation and peritumoral edema, resulting in radicular pain, motor deficits, and sensory disturbances, which was often treated through surgical resection (7, 8).

Schwannomas are slow-growing benign neoplasms that typically exhibit non-invasive behavior towards surrounding tissues. Consequently, they manifest as well-encapsulated masses with distinct boundaries on ultrasonography and demonstrate an absence or minimal presence of intra-tumoral blood flow signals on Color Doppler flow imaging (CDFI) (9, 10). Given their propensity for growth along nerve trunks, schwannomas may demonstrate compressed or encapsulated nerve fibers at the tumor’s poles, a sonographic feature known as the “tail sign”, which is considered characteristic for the diagnosis of schwannomas (11). However, the detection rate of this classic sign in clinical practice is relatively low, potentially due to early-stage disease where nerve compression is subtle or obscured by adjacent large vessels or lymph nodes (12–14). It is important to note that some schwannomas may present solely as cervical masses, necessitating differentiation from other neck lesions such as lymphomas, metastatic tumors, neurofibroma, ganglioneuroma, meningioma, hemangioma or non-neoplastic lesions (e.g., abscess or hematoma). Cervical lymphomas typically manifest as multifocal lesions with a fine reticular internal pattern on ultrasound and abundant blood flow on CDFI (15, 16). The current lesion is solitary, avascular, and cystic-solid nature and coupled with the lack of systemic symptoms (e.g., fever, weight loss), making rendered lymphoma unlikely. Meanwhile, metastatic tumors appear as heterogeneously hypoechoic masses due to variable tissue composition from different primary sources. They may exhibit irregular borders and infiltrative growth patterns, which can be indicative of aggressive behavior. CDFI are often more heterogeneous compared to schwannomas due to the diverse vascular supply. Generally, diagnosis often relies on the patient’s history of a known primary malignancy and the presence of other lesions that suggest dissemination (17, 18). The absence of a known primary malignancy and the well-demarcated, encapsulated appearance in this case argued against metastasis. Neurofibromas, another common peripheral nerve sheath tumor, may exhibit similar imaging features to schwannomas, including a dumbbell-shaped morphology. However, neurofibromas typically lack cystic degeneration and are unencapsulated, with a more infiltrative growth pattern on histopathology. Unlike schwannomas, neurofibromas demonstrate CD34 protein positive rather than SOX-10 strong peripheral staining seen in schwannomas (19, 20). Ganglioneuromas arise from sympathetic ganglia and are characterized by elongated spindle cells and mature ganglion cells. They often display a paravertebral location but lack the cystic components and nerve root origin seen in the current case (21–23). Spinal meningiomas is rare in the cervical region and often present as intradural extramedullary masses with dural attachment. And meningiomas typically show homogeneous enhancement post-contrast and may exhibit calcifications on MRI/CT scan, the mentioned features are absent in this case (24–26). Hemangiomas often show irregular or infiltrative borders with a “honeycomb” or lattice-like pattern due to vascular channels and fatty stroma on ultrasound imaging. And they may deform under transducer pressure due to their vascular nature and usually demonstrate marked vascularity with a “peripheral-to-central” flow pattern on CDFI (27–29). The absence of these features, along with the tumor’s nerve root origin, excluded hemangiomas in this case. Notably, some non-neoplastic lesions, such as abscess or hematoma, may mimic cystic schwannomas. However, these non-neoplastic lesions often accompanied with typical clinical history (fever, trauma, or acute onset) and imaging findings (perilesional edema or fluid-debris levels), lacking of mentioned features helped rule out these entities (30–32).

In the present case, the dumbbell-shaped schwannoma, which grew from the C5 intervertebral foramen into the paravertebral region, did not exhibit the classic “tail sign” on ultrasound imaging, potentially due to extensive local invasion and obscuration by osseous structures. Notably, the presence of cystic degeneration, a feature that is considered pathognomonic for schwannomas, was observed. In fact, only a mixed cystic and solid mass measuring 3 cm × 2 cm in the right supraclavicular region was identified during the initial ultrasound examination. Further inquiring about patient’s symptoms of right-hand pain, the sonographer to use an abdominal probe for further investigation, ultimately discovering and diagnosing this large dumbbell-shaped schwannoma (6.07 cm × 2.54 cm) that extended from the intervertebral foramen into the paravertebral area. Subsequently, the patient underwent magnetic resonance imaging (MRI) to delineate the anatomical relationship between the tumor and surrounding neural, vascular, and osseous structures, followed by the surgical tumor resection. The surgical challenge in treating dumbbell-shaped cervical schwannomas lies in the resection of the extravertebral component of the tumor, given the complexity of the surrounding structures and proximity to vital vascular and neural tissues such as the vertebral artery. The specific surgical approach must be individually selected based on the surgeon’s experience and the growth characteristics of the tumor. Typically, tumors with the majority of their mass within the spinal canal (Azsazuma classification types I, IIa, IIIa, and IV) can be managed with a posterior approach alone (33, 34). Otherwise, if the tumor extends more than 4 cm beyond the spinal canal or completely encircles the vertebral artery, making it unresectable in the initial phase, a later resection through a lateral approach may be necessary to remove residual tumor (35, 36). In this case, the cervical dumbbell tumor extended more than 4 cm across the midline, involving the interscalene space and deep cervical fascia, making it difficult to fully visualize and excise the lateral boundary of the tumor through a posterior midline approach alone. Therefore, the surgeons opted for a two-stage procedure, with the first surgery being a posterior approach to remove the intradural component, followed by a lateral approach to excise the extra-dural component. Pathological results confirmed the tumor to be a classic schwannoma, with positive expression of S-100 and SOX-10 proteins and negative expression of epithelial membrane antigen. Gratifyingly, at the 6-month and 1-year postoperative follow-up, the patient’s symptoms, including hand numbness and neck pain, had completely resolved. Furthermore, the absence of residual or recurrent tumor on MRI signifies a successful curative outcome (**Figure 3**).

This study has several limitations. Firstly, while demonstrating diagnostic value in this case, ultrasonography is constrained by technical challenges that osseous structures restrict its penetration and resolution, potentially obscuring intraspinal or deeply situated tumor components. Secondly, recognizing of sonographic features (e.g., “tail sign” or cystic degeneration) is highly operator-dependent, requiring specialized expertise, and atypical schwannomas (e.g., purely solid masses mimicking lymphomas or metastases) risk misdiagnosis without confirmatory MRI or biopsy. Furthermore, the reviewed literature predominantly focused on surgical outcomes and imaging modalities, with limited emphasis on long-term functional recovery or cost-effectiveness analyses of diagnostic pathways. Finally, the postoperative follow-up period of one year, while sufficient to confirm tumor resection, may be insufficient to assess long-term recurrence risks or late complications, particularly for schwannomas with atypical features.

In summary, this study underscores ultrasonography could be prioritized as the initial evaluation of spinal tumors due to its rapid, non-invasive, and accessible in delineating soft tissue lesions, particularly for patients with palpable cervical masses and neurological symptoms. Furthermore, dumbbell-shaped tumors involving both intraspinal and extra-spinal compartments necessitate a multidisciplinary approach to formulate staged surgical strategies, aimed at minimizing intraoperative risks to critical neurovascular structures, while ensuring complete tumor resection.

## Data Availability

The raw data supporting the conclusions of this article will be made available by the authors, without undue reservation.
